# Robotic single‐port surgery: Preliminary experience in general surgery

**DOI:** 10.1002/rcs.2453

**Published:** 2022-08-19

**Authors:** Francesco M. Bianco, Nicolas H. Dreifuss, Betty Chang, Francisco Schlottmann, Antonio Cubisino, Alberto Mangano, Yevhen Pavelko, Mario A. Masrur, Pier C. Giulianotti

**Affiliations:** ^1^ Division of General Minimally Invasive, and Robotic Surgery Department of Surgery University of Illinois at Chicago Chicago Illinois US

**Keywords:** cholecystectomy, Da Vinci SP, inguinal hernia, robotic surgery, single port surgery, single‐incision surgery

## Abstract

**Background:**

We aim to analyse the safety and feasibility of the DaVinci Single Port (SP) platform in general surgery.

**Methods:**

A prospective series of robotic SP transabdominal pre‐peritoneal inguinal hernia repairs (SP‐TAPP) and cholecystectomies (SP‐C) (off‐label) were analysed. Primary endpoints were safety and feasibility defined by the need for conversion and incidence of perioperative complications.

**Results:**

A total of 225 SP procedures were performed; 84 (37.3%) SP‐TAPP (70 unilateral, 7 bilateral), and 141 (62.7%) SP‐C. There were no conversions or additional ports placed. Mean console time was 17.6, 31.9, and 54 min for SP‐C, unilateral, and bilateral SP‐TAPP, respectively. There was no mortality, intraoperative or major postoperative complications. Mean LOS was 2.7 h for elective SP‐TAPP and 2.3 h for SP‐C.

**Conclusion:**

Robotic SP surgery is safe and feasible for two of the most performed general surgery operations. Further experience might allow expanding the applications of robotic single‐incision surgery for other procedures.

## 1 INTRODUCTION

As part of the minimally invasive surgery revolution, single incision laparoscopic surgery (SILS) represented a step forward in the direction of reducing invasiveness and surgical trauma. SILS has demonstrated to be a safe and feasible alternative for both cholecystectomies and inguinal hernia repairs.^1^, ^2^, ^3^, ^4^, ^5^ Reduced postoperative pain, recovery time, improved cosmesis and body image were some of the reported advantages of SILS when compared to standard multiport laparoscopy.^5^, ^6^, ^7^, ^8^, ^9^, ^10^, ^11^ However, SILS never achieved large popularity in the surgical community due to technical limitations such as a reduced ability to triangulate, internal and external clashing, and ergonomic discomfort.^12^ In 2011, specialised instruments for the Da Vinci surgical system (Da Vinci Single‐Site) were developed to perform robotic‐assisted single‐incision procedures.^13^ Although this technology improved some of the technical constraints of traditional SILS, the instrument's excessive flexibility, lack of endowrist, and limited strength prevented its widespread adoption.

Recently, a completely redesigned single port (SP) robotic platform has been specifically developed for single‐incision surgery and reignited the interest in the approach as it carries potential to overcome many of the above‐mentioned limitations and allow a wider range of surgical applications. This platform provides the surgeon with similar capabilities as the DaVinci multiport platform, with the exception that 3 multi‐jointed, wristed instruments and a 3D‐HD articulating scope are introduced through a SP. This improved technology allows distal instrument triangulation, excellent internal and external range of motion, and 360°multi‐quadrant access through a single 2.5 cm skin incision. The DaVinci SP has already been FDA‐approved for transoral endoscopic head and neck surgery and urology with promising results.^14^, ^15^

In this manuscript, we report the first results of the DaVinci SP platform applications in general surgery (SP transabdominal pre‐peritoneal inguinal hernia repair [SP‐TAPP] and SP cholecystectomy [SP‐C]) conducted under an IRB approved protocol.

## 2 MATERIALS AND METHODS

### 2.1 Da Vinci single port technology

**FIGURE 1:**
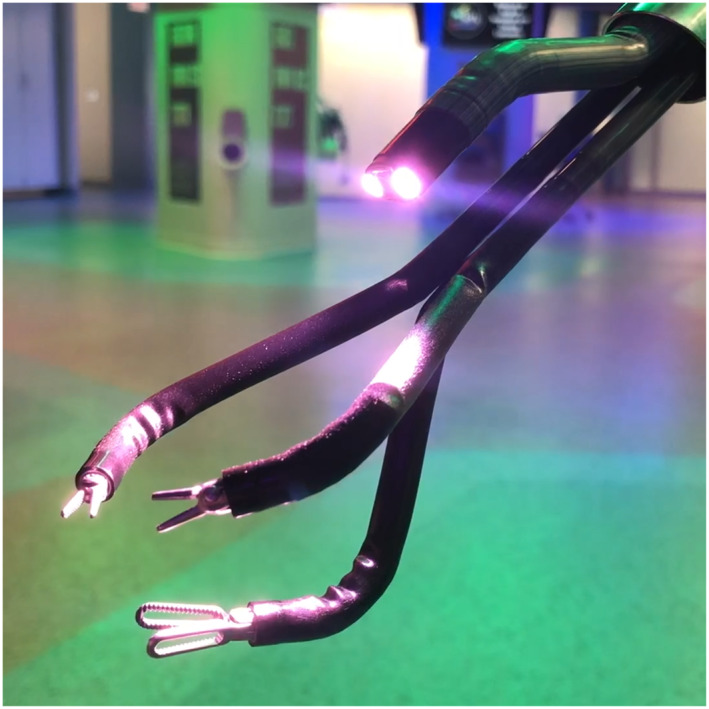
Robotic single port camera and instruments

The surgical system used was the DaVinci SP, SP 1098 Surgical System (Intuitive Surgical Inc, Sunnyvale, California). This system is composed of a surgeon console, a patient‐side cart, and a vision cart. The surgeon's console has two 3D‐HD screens and a tridimensional superimposed image which functions as an instrument guidance system that tracks the location of the robotic port, camera, and the instruments in real time during the procedure. The patient‐side cart is equipped with a single arm with four instrument drives that control the 12 × 9 mm articulating camera and three 6‐mm double‐jointed articulating endowristed instruments. The instruments are connected to the SP arm drive in a similar fashion to the Da Vinci Xi platform. Afterwards, they are introduced into the abdominal cavity through a single metal cannula. The cannula has an entry guide with one oval lumen (9 mm, for the scope) and three circular lumens (6 mm, for the instruments). The camera and the instruments are introduced in the abdominal cavity through a 25 mm multichannel port (Figure 1). The system is provided with a 3D high definition fully wristed endoscope. The robotic arm can be manipulated independently of the individual robotic instruments. These features allow virtually 360° anatomical (multi‐quadrant) access through the fulcrum of the SP. The available instruments for the SP platform include cadiere forceps, round tooth retractor, medium‐large clip applier, needle driver, fenestrated bipolar forceps, maryland bipolar forceps, monopolar curved scissors, monopolar cautery hook, and monopolar cautery spatula. The hand controllers of the Da Vinci SP system are the same as the previous multiport Da Vinci platforms. The instruments are also controlled in a similar way. The camera is controlled differently (due to its articulation). The scope clutch activates three different camera modes. The adjust mode, which allows to move the camera and navigate in the workspace while maintaining the instruments in the same position; the camera control mode, which allows to move the camera and the joints without moving the instruments; and the relocate mode, which allows to reposition the camera and the instruments simultaneously by moving the entire instrument cluster.

### 2.2 Study design and population

This study was conducted under an Institutional Review Board (IRB) approved protocol (IRB #2021‐0520). A review of a prospectively collected database of all patients who underwent single‐incision surgery with the Da Vinci SP surgical system from July 2019 to September 2021 was performed. This article was written following the standards of the STROBE guidelines for observational studies.^16^

During the study period, procedures performed with the SP platform included: cholecystectomy, TAPP inguinal hernia repair, ventral/incisional hernia repair, Nissen fundoplication, and partial gastric resection. SP‐C and SP‐TAPP were included in the analysis.

The surgical procedures reported in this article were performed by one surgeon (FMB) with previous experience in robotic multiport and Da Vinci single site surgery. Before starting the SP cases on humans, training was completed on the SP platform using simulation, 2 days dry and wet laboratories, and case observation. For the first three cases, an internal proctor from the Urology department with SP experience was present. All the nursing staff and scrub techs involved had previous experience with the system. All procedures were assisted by general surgery residents. A senior resident who also received training in the laboratory was present for the first two cases. The senior resident was shadowed by a junior resident who eventually took over the following cases. All new residents were proctored by a previously trained resident before assisting autonomously.

Case selection (elective cases in patients without super‐obesity or previous abdominal operations) was performed for the first 15 procedures (cholecystectomies). After that, all cholecystectomies were booked SP. SP‐C was indicated for symptomatic cholelithiasis, acute cholecystitis, chronic cholecystitis, porcelain gallbladder, gallbladder polyps, choledocholithiasis, and gallstone pancreatitis. Patients with suspected choledocholithiasis underwent preoperative magnetic resonance cholangiography and if positive, a preoperative endoscopic retrograde cholangiopancreatography (ERCP) for bile duct stones removal was done. After the first 20 cholecystectomies we started performing inguinal hernia repairs. SP‐TAPP was indicated for femoral, unilateral, and bilateral inguinal hernias. Similarly, during the first 10 cases inguinoscrotal, recurrent hernias (laparoscopically approached) and/or patients with previous prostatectomy were avoided. After the first 10 cases, all inguinal hernias were enroled except large inguinoscrotal hernias with chronically incarcerated bowel. After the first 120 cases, we started to include selected gastric resections, hiatal hernias, and ventral hernias.

The procedure and the innovative nature of the approach were explained to the patients, along with the expected outcomes and potential risks. Moreover, the patients were informed about the alternative approaches (laparoscopic and robotic multiport) before written consent was given.

Follow‐up was performed in the office on postoperative week 2 and with phone calls at variable intervals. A modified version of the PINQ telephone questionnaire (previously validated for inguinal hernia recurrence detection) was used to screen inguinal hernia recurrences and umbilical port incisional hernias.^17^ If the telephone screening was positive, patients were scheduled for an in‐persons physical examination at the office. Moreover, satisfaction with cosmetic results were addressed during the follow‐up calls by a 1 (unsatisfied) to 10 (extremely satisfied) scale.

### 2.3 Procedure details

The operation starts by creating a single access through a vertical 2.5 cm skin incision immediately lateral to the umbilicus. The subcutaneous space is dissected bluntly and with monopolar energy. The fascia and peritoneum are opened, and an army navy retractor is used to lift‐up the wound while the single‐port is advanced horizontally gently stretching the fascial incision. The dissection starts after the pneumoperitoneum is established, the robot is docked, and the articulated camera and 3 double‐jointed instruments are connected to the single arm.

#### 2.3.1 SP cholecystectomy

**FIGURE 2:**
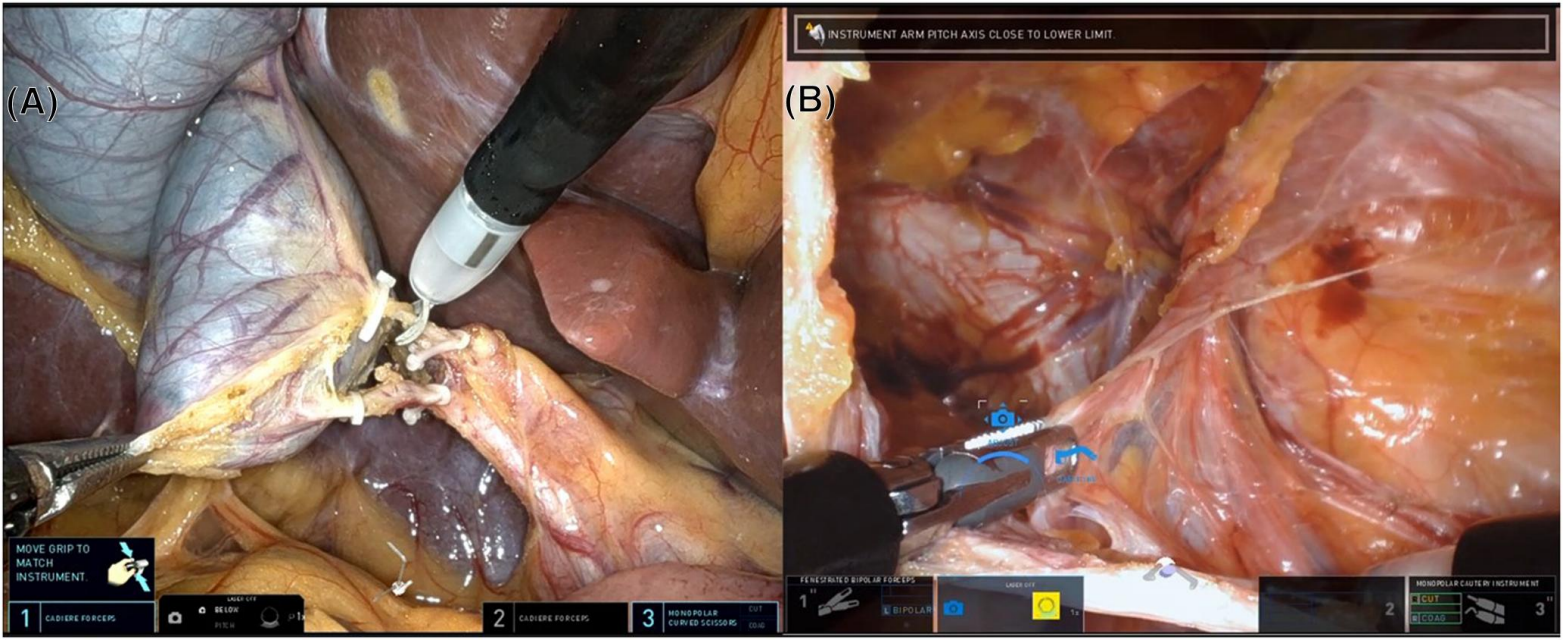
Intraoperative pictures: Single Port cholecystectomy (A) and Single Port transabdominal preperitoneal (TAPP) inguinal hernia repair (B)

The gallbladder is retracted cephalad using a cadiere forceps in the third robotic arm reaching from the top of the port. The gallbladder infundibulum is retracted laterally using a bipolar forceps and the Calot's triangle is dissected with the monopolar hook. The cystic duct and artery are identified, skeletonised, and divided between hem‐o‐lok clips (Figure 2A). The gallbladder is detached from the liver bed with the robotic monopolar hook and extracted within the SP device. In cases of acute cholecystitis and difficult retraction, the gallbladder is decompressed with a suction device (argyle suction catheter) which is inserted through the instrument port or between the skin and the SP canula. In case of infection or perforation, the gallbladder is retrieved in a 5‐mm endobag introduced through one of the robotic ports.

#### 2.3.2 SP TAPP inguinal hernia repair

The peritoneum is incised with the monopolar hook to access the preperitoneal space. The hernia sac is reduced, and the lower epigastric vessels and elements of the spermatic cord or round ligament are identified (Figure 2B). The bipolar forceps is used for retraction and haemostasis and the hook for electrocautery dissection. Once the preperitoneal space is fully dissected, a 3D BardTM mesh is introduced through the SP and fixed immediately above the Cooper's ligament. Finally, the peritoneum flap is closed with a running absorbable suture. In some cases (short‐torso patients) a floating dock is used to facilitate the flap closure, as a minimum distance of 10 cm from the target anatomy is required to fully deploy the instruments inside the abdominal cavity. Floating dock is obtained placing an Alexis wound retractor and tying a suture around the retractor and the port.

**FIGURE 3:**
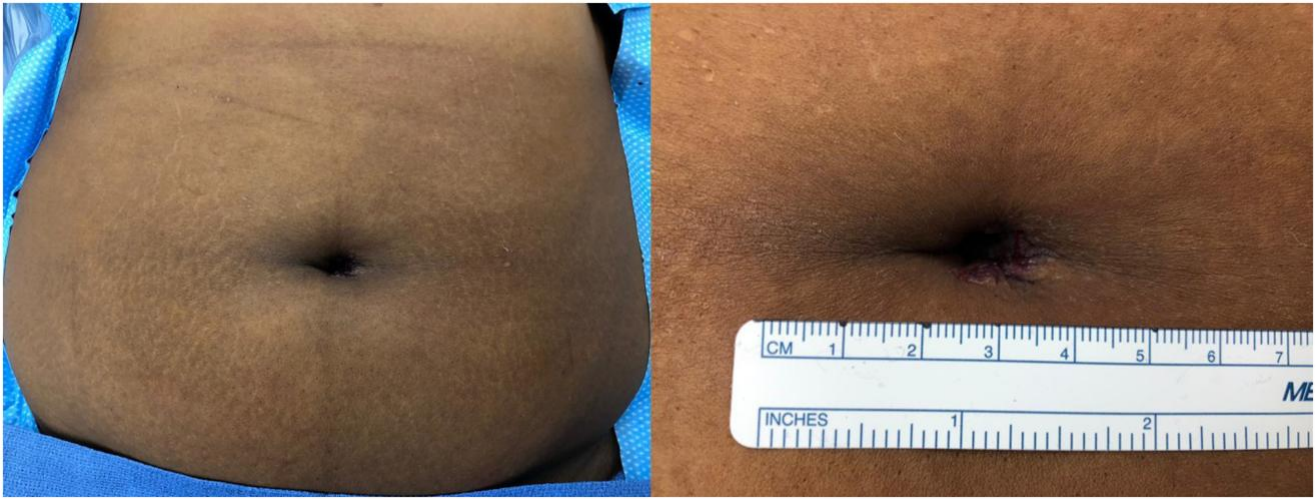
Umbilical incision closure

At the end of the procedure, the SP device is removed, and the fascia defect is closed with figure‐of‐eight polydioxanone 1 sutures. The subcutaneous space is closed with vycril sutures and the skin with subcuticular interrupted sutures of monocryl 4–0 (Figure 3). The same closure technique was used after cholecystectomies and inguinal hernia repairs.

### 2.4 Variables and outcomes

Perioperative information was collected using standardized case report forms and entered prospectively into an institutional database. Data collected included age, gender, body mass index (BMI), American Society of Anaesthesiologists (ASA) classification, presence of comorbidities, previous abdominal surgeries, and hernia repairs. Perioperative variables such as hernia type (according to Nyhus classification), associated procedures, intraoperative complications, conversion (to multiport laparoscopy or to open surgery), additional port placement rate, and blood loss were also registered. Operative time metrics included: skin incision to port placement time, port placement to docking start time, docking time (time necessary to dock the robot and connect the required instruments to start the operation), end of docking to first instrument movement time, time to first clip (interval time between the first instrument movement and the first cystic clip application), first clip‐gallbladder detached time (interval time between the application of the first cystic clip and full dissection of the gallbladder from liver's bed), time to mesh placement (interval time between the first instrument movement and deployment), mesh placement‐flap closure time (time necessary to close the peritoneal flap), console time, undocking time, undocking to fascia closure start time, fascia closure time, time from skin incision to fascia closure, skin closure time, and total operative time. Recovery parameters such as recovery time, length of hospital stay (LOS), overall 30‐day morbidity (according to Clavien‐Dindo classification), major morbidity (defined as Clavien‐Dindo ≥3a), urgent reoperations, 30‐day readmission, inguinal recurrence, and umbilical port incisional hernia rates were also considered for analysis.

Primary endpoints were safety and feasibility defined by the need of conversion and incidence of perioperative complications. Secondary endpoints included mean operative time, console time, length of hospital stay (LOS), and port‐site incisional hernia rate.

## 3 STATISTICAL ANALYSIS

For descriptive statistics, continuous data were summarised by reporting mean, median, range, and standard deviation. Categorical data were summarised using frequency and percentage.

## 4 RESULTS

**FIGURE 4:**
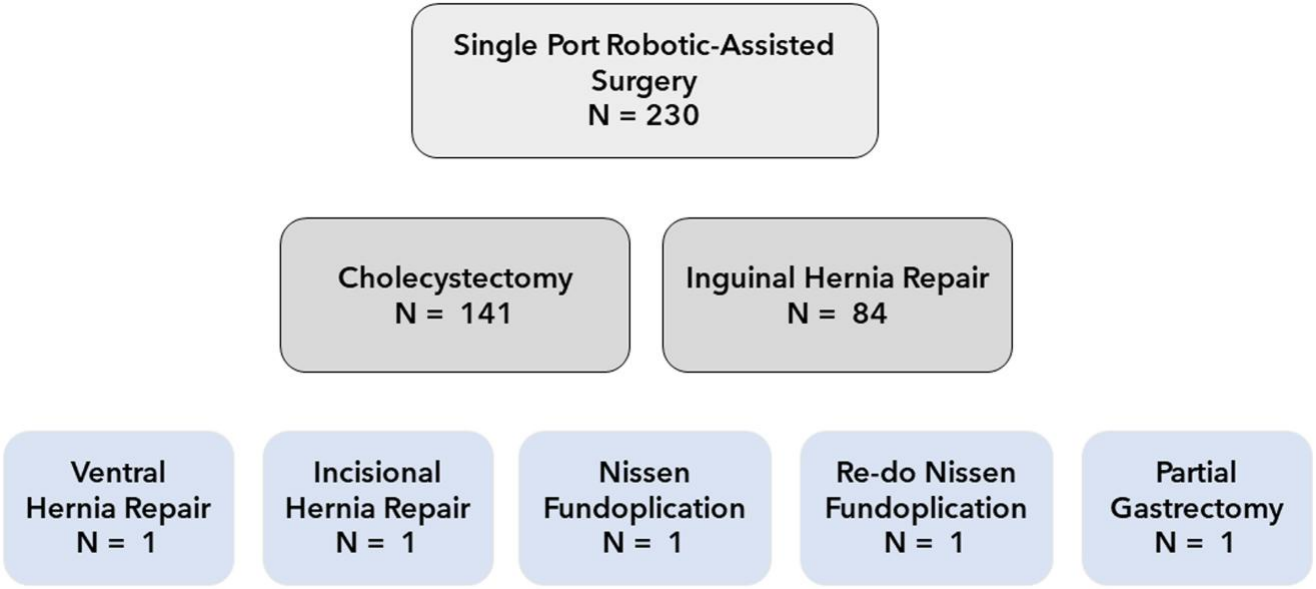
Cases performed with the robotic single port platform

During the study period, 222 patients underwent 230 robotic SP operations. Procedures performed included 141 SP‐C and 84 SP‐TAPP. The remaining four patients underwent a SP partial gastrectomy, hiatal hernia repair with Nissen fundoplication, ventral hernia repair, and an incisional hernia repair and were excluded from the analysis (Figure 4).

Demographics and preoperative variables are shown in Table 1. In the SP‐TAPP group, the mean age was 52.1 years and 93.5% of patients were male. Most patients were ASA class 1–2 (80.5%), and the mean BMI was 27.3 (19.5–41.6) kg/m^2^. In the SP‐C cohort, 74.5% of patients were female with a mean age of 41 years. Most patients (72.4%) had low anesthesiologic risk (ASA 1–2). Obesity (BMI > 30 kg/m^2^) was present in 62.4% of the patients and in this sub‐group the average BMI was 38.8 kg/m^2^. Moreover, 48 (34%) patients from the SP‐C group and 20 (26%) patients from the SP‐TAPP group had previous abdominal surgeries.

**TABLE 1:** Demographics and preoperative variables

	SP‐TAPP	SP‐C
	*n* = 77	*n* = 141
Gender		
Female, *n* (%)	5 (6.5)	105 (74.5)
Male, *n* (%)	72 (93.5)	36 (25.5)
Mean age, years (range)	52.1 (15–80)	41 (18–85)
Mean BMI, kg/m^2^ (range)	27.3 (19.5–41.6)	33.9 (14.8–71.8)
ASA, *n* (%)		
I	17 (22.1)	19 (13.5)
II	45 (58.4)	83 (58.9)
III	15 (19.5)	38 (26.9)
IV	0 (0)	1 (0.7)
Smokers, *n* (%)	33 (42.8)	41 (29.1)
Hypertension, *n* (%)	29 (37.7)	51 (36.2)
Diabetes mellitus, *n* (%)	10 (13)	25 (17.7)
Chronic kidney disease, *n* (%)	4 (5.2)	13 (9.2)
Respiratory disease, *n* (%)	15 (19.5)	29 (20.6)
Previous abdominal surgery, (%)	20 (26)	48 (34)
Supramesocolic	5 (6.5)	7 (5)
Inframesocolic	15 (19.5)	41 (29)

Abbreviations: ASA, American society of anaesthesiologist classification; BMI, body mass index; SP‐C, Single port cholecystectomy; SP‐TAPP, Single port transabdominal preperitoneal inguinal hernia repair.

Indications for SP‐C included 101 (71.6%) symptomatic cholelithiasis, 27 (19.1%) acute cholecystitis, 3 (2.1%) chronic cholecystitis, 3 (2.1%) gallbladder polyps, 2 (1.4%) choledocholithiasis, 2 (1.4%) gallstone pancreatitis, 2 (1.4%) porcelain gallbladder, and 1 (0.7%) biliary dyskinesia.

SP‐TAPP was indicated for 69 (89.6%) inguinal unilateral (43 right and 26 left), 7 (9.1%) inguinal bilateral, and 1 (1.3%) femoral unilateral hernia.

Mean skin incision to port placement was 5.4 min for SP‐TAPP and 4.7 min for SP‐C. Mean docking time was 2.3 and 2.4 min for SP‐TAPP and SP‐C, respectively. Mean console time for unilateral and bilateral inguinal hernia repairs was 31.9 and 54 min, respectively. Mean operative time was 79.1 min in unilateral and 111.7 in bilateral inguinal hernia repairs. In SP‐C, the mean console time was 17.6 min, and the mean operative time was 65.5 min. Additional operative time metrics can be found in Table 2.

**TABLE 2:** Operative time metrics

	SP‐TAPP	SP‐C
	*n* = 77	*n* = 141
Skin incision to port placement, minutes		
Mean (SD)	5.4 (3.8)	4.7 (3.3)
Median	4	4
Range	1–24	1–7
Port placement to docking start, minutes		
Mean (SD)	3 (1.8)	4 (3.3)
Median	3	4
Range	1–8	1–17
Docking time, minutes		
Mean	2.3 (2)	2.4 (1.9)
Median	2	2
Range	1–10	1–18
Docking end to first instrument movement, minutes		
Mean	2.6 (2.4)	2.5 (2.3)
Median	2	2
Range	0–12	0–16
Time to first clip, minutes	‐	
Mean (SD)		7 (6.9)
Median	‐	5
Range	‐	3–55
Time first clip—GB detached, minutes		
Mean (SD)	‐	10.6 (8.5)
Median	‐	9
Range	‐	4–73
Time to mesh placement, minutes		
Mean (SD)	18.1 (10.7)	‐
Median	17	‐
Range	7–36	‐
Mesh placement‐flap closure time, minutes		
Mean (SD)	15.4 (8.5)	‐
Median	15	‐
Range	5–36	‐
Console time, minutes		
Mean (SD)	31.9 (14.4)	17.6 (13.5)
Median	33	14
Range	15–55	6–99
Undocking time, minutes		
Mean (SD)	4.1 (5.6)	2.4 (2.3)
Median	2	2
Range	1–26	1–14
Undocking to fascia closure start time, minutes		
Mean (SD)	3.1 (2.2)	3.7 (2.4)
Median	2	3
Range	1–10	0–18
Fascia closure time, minutes		
Mean (SD)	12.3 (7.5)	9.9 (4.6)
Median	9	9
Range	5–19	2–32
Skin incision to fascia closure time, minutes		
Mean (SD)	65.3 (20.1)	50.7 (28)
Median	67	43
Range	39–118	26–173
Skin closure time, minutes		
Mean (SD)	13.4 (10.9)	14.9 (10.1)
Median	13	14
Range	2–33	4–30
Operative time, minutes		
Mean (SD)	79.1 (35.5)	65.5 (28.7)
Median	84.5	60
Range	45–119	36–177

Abbreviations: GB, gallbladder; SP‐C, Single port cholecystectomy; SP‐TAPP, Single port transabdominal preperitoneal inguinal hernia repair.

An associated procedure was performed in 18 (23.4%) of SP‐TAPP (12 umbilical hernias, 2 prostatectomies, 2 hydroceles, 1 partial nephrectomy, and 1 ventral hernia repair) and 7 (5%) of SP‐C (4 umbilical hernia, 1 partial nephrectomy, 1 ventral hernia repair, and 1 liver biopsy).

There were no intraoperative complications, conversions, or additional ports placed (excluding combined cases with urology) in the series (Table 3). The first assistant was a postgraduate year (PGY) 1 resident in 19%, PGY‐2 in 18.4%, PGY‐3 in 28.4%, PGY‐4 in 21.6%, and PGY‐5 in 12.6% of the cases.

**TABLE 3:** Operative variables

	SP‐TAPP	SP‐C
	*n* = 77	*n* = 141
Associated procedure, *n* (%)	18 (23.4)	7 (5)
System errors, *n* (%)	2 (2.6)	3 (2.1)
Recoverable fault	2 (2.6)	1 (0.7)
Camera issue	0 (0)	1 (0.7)
Sterile adaptor error	0 (0)	1 (0.7)
Additional port, *n* (%)	0 (0)	0 (0)
Conversion, *n* (%)	0 (0)	0 (0)
Intraoperative complications, *n* (%)	0 (0)	0 (0)

Abbreviations: SP‐C: Single port cholecystectomy, SP‐TAPP: Single port transabdominal preperitoneal inguinal hernia repair.

Most of the operations (SP‐TAPP: 97.4%, SP‐C: 80.1%) were performed in an outpatient basis with a mean recovery time of 2.7 and 2.3 h for the SP‐TAPP and SP‐C groups, respectively. The two admitted patients in the SP‐TAPP cohort were the combined cases with the urology team (partial nephrectomy and prostatectomy). In the SP‐C group, 28 patients did not undergo same‐day discharge: 26 were previously admitted due to acute presentations (cholecystitis, suspicious of common bile duct stones, pancreatitis), and 2 were admitted postoperatively due to persistent nausea and vomiting.

Overall 30‐day morbidity was 6.5% in the SP‐TAPP group and 1.4% in the SP‐C group. All the complications were minor (Clavien I‐II) and included 3 seromas (resolved spontaneously), 2 prolonged postoperative ileus (managed conservatively), 1 urinary retention (required foley catheter), and 1 urinary tract infection (antibiotic treatment). There were no major complications, urgent reoperations, or mortality. Three patients were readmitted (1.4%), 2 for prolonged postoperative ileus (1 SP‐C and 1 SP‐TAPP), and 1 for a urinary retention (SP‐C) (Table 4).

**TABLE 4:** Postoperative outcomes

	SP‐TAPP	SP‐C
	*n* = 77	*n* = 141
Same‐day discharge, *n* (%)	75 (97.4)	113 (80.1)
Recovery time, minutes (range)	164.5 (40–352)	136.5 (43–291)
Admitted preoperatively, *n* (%)	0 (0)	26 (18.4)
Admitted postoperatively, *n* (%)	2 (2.6)	2 (1.4)
Mean LOS, days (range)	0 (0–2)	0.3 (0–4)
30‐day overall morbidity, *n* (%)	5 (6.5)	2 (1.4)
Clavien‐Dindo, *n* (%)		
I‐II	5 (6.5)	2 (1.4)
	3 seromas	1 ileus
	1 ileus	1 urinary retention
	1 UTI	
III	0 (0)	0 (0)
IV	0 (0)	0 (0)
V	0 (0)	0 (0)
30‐day readmissions, *n* (%)	1 (1.3)	2 (1.4)
	1 ileus	1 ileus
		1 urinary retention
Mean follow‐up, months (range)	8.8 (1–27)	8.9 (1–28)
Inguinal recurrence, *n* (%)	0 (0)	‐
Port site incisional hernia, *n* (%)	1 (1.3)	2 (1.4)

Abbreviations: LOS, length of hospital stay; SP‐C, Single port cholecystectomy; SP‐TAPP, Single port transabdominal preperitoneal inguinal hernia repair.

After a mean follow‐up of 8.9 months, there were 3 (1.4%) port‐site hernias and no inguinal recurrences. Mean satisfaction with scar cosmesis was 9.2 (4–10).

## 5 DISCUSSION

Since Muhe's introduction in 1985, laparoscopic cholecystectomy has been the treatment of choice for gallbladder disease.^18^ Similarly, laparo‐endoscopic techniques are now one of the preferred approaches for inguinal hernia repairs.^19^ To further reduce operative trauma, SILS was developed. The proposed advantages of single‐incision over multiport laparoscopy include a reduced risk of wound infection, less postoperative pain, faster recovery, improved cosmesis, and body image.^5^, ^6^, ^7^, ^8^, ^9^, ^10^, ^11^ For instance, the multicenter double‐blinded SPOCC‐trial randomized 110 patients to SILS cholecystectomy (SILS‐C) and laparoscopic multiport cholecystectomy (LMC). SILS‐C resulted in better short‐term and long‐term cosmesis and body image, reduced postoperative pain, and improved quality of life with similar LOS and complication rates.^7^ On the contrary, concerns have been raised regarding the steeper learning curve, prolonged operative time, and decreased visualisation/exposure of critical structures. These might result in a higher risk of serious complications such as bile duct injuries.^20^ Moreover, Ma and colleagues reported that an additional 3 mm instrument was necessary in 66.6% of SILS‐C to properly retract the gallbladder and the operative time of SILS doubled (SILS‐C: 88 vs. LMC: 44 min, *p* < 0.05) the multiport technique.^21^ In our series of SP‐C, no additional ports or extra‐corporeal sutures to retract the gallbladder were required to complete the procedure.

SILS has also been proved safe and feasible for inguinal hernia repairs.^4^, ^5^, ^22^, ^23^ The randomized controlled trial by Cardinali et al compared 200 totally extraoperitoneal inguinal hernia repairs with the multi‐trocar or SILS approach.^4^ The authors found similar outcomes regarding postoperative pain, length of stay, overall morbidity, and recurrence rates after 2 years of follow‐up. However, operative time was shorter with the multiport approach (SILS: 50.9 vs. multiport: 44.9, *p* = 0.01) and cosmetic satisfaction was higher with SILS repairs (SILS 7.5 vs. multiport: 6.9, *p* = 0.003). A recent meta‐analysis of 16 studies that compared SILS with laparoscopic multiport inguinal hernia repairs, found that both approaches were equivalent regarding postoperative outcomes.^23^ Despite SILS‐C and SILS inguinal hernia repair have proven to be safe and feasible, inherent difficulties of the surgical technique, steeper learning curve, cost‐effectiveness concerns, and dubious advantages limited the broad adoption of these techniques.

In 2011, new accessories and instruments for single‐incision surgery were developed for the robotic platform (Da Vinci Single‐Site). Several authors published their experience with favourable outcomes, some of which showed advantages over the classic SILS technique.^13^, ^24^, ^25^, ^26^ For instance, the randomized trial performed by Grochola et al found similar postoperative morbidity, reduced surgeon's mental, physical stress load, and shorter LOS in patients undergoing robotic single‐site cholecystectomy when compared to SILS approach counterparts.^24^ Similarly, a recent comparative study showed lower rates of gallbladder perforation and bile spillage with the single‐site approach.^25^ Despite all, the higher costs, technical drawbacks (external clashing, lack of endowrist), and lack of significant clinical outcomes benefits made the use of this approach questionable.^27^

In 2018, a robotic platform specifically designed for SP surgery was released. The technical improvements of the Da Vinci SP system (multi‐jointed instruments and scope, multi‐quadrant access, navigation system, lack of external clashing) reignited the interest in the single incision approach. Up to now, this platform has been FDA‐approved for urological operations and transoral endoscopic robotic surgery (TORS) for head and neck cancers, with promising outcomes and a fast learning curve.^14^, ^15^ Despite not being yet approved for general surgery procedures in the US, a few cases have been described in the literature.^28^, ^29^, ^30^, ^31^

To our knowledge, our series represents the largest clinical experience with robotic SP cholecystectomies and the first report on inguinal hernia repairs. The absence of conversion, need for additional ports, intraoperative and major postoperative complications proved the feasibility and safety of the approach. Minor complication rates in SP‐C and SP‐TAPP are within the reported for the gold standard approach (multiport laparoscopy).^32^, ^33^, ^34^ Mean operative and console time from previous reports on SILS and robotic single‐site cholecystectomies ranged from 71 to 101.6 min and 32–53 min, respectively.^7^, ^13^, ^20^, ^21^, ^24^, ^25^, ^27^, ^35^, ^36^ Interestingly, using the new SP robotic platform we found a shorter mean operative time (65.5 min) and console time (17.6 min). Mean operative time for unilateral SP‐TAPP was within the reported range in the literature (38.7–91.2 min) for SILS and robotic single‐site TAPP repairs.^4^, ^22^, ^23^, ^26^, ^37^ It is worth to mention, that a significant amount of the non‐console time is spent in the subcutaneous tissue and skin closure which is usually performed by medical students at our institution.

Mean LOS after SP‐TAPP and SP‐C were 2.7 and 2.3 h, respectively. These were lower than reported by previous series of SILS and robotic single‐site approaches.^4^, ^7^, ^22^, ^25^, ^26^, ^27^, ^35^, ^37^ Unlike previous studies on SILS and robotic single site, most of our cases (>75%) were performed without patient selection including emergent indications, recurrent hernias, patients with previous abdominal operations, and super‐obesity (BMI up to 71.8 kg/m^2^).

A potential drawback of single‐incision procedures is the risk of incisional hernias due to the larger fascial incision required for the access. Weiss et al evaluated wound complications in 1145 SILS procedures, and after a median follow‐up of 22.1 months, 2.5% of wound complications and 1.4% of incisional hernias were recorded.^38^ Similarly, we found 1.4% of port‐site incisional hernias in our series. The average length of follow‐up is still too short to be able to compare long‐term outcomes. However, at this point, these rates are similar to those reported for conventional laparoscopy (up to 5.2% of trocar site hernias).^39^, ^40^ Conversely, other authors reported a higher risk of incisional hernia with single‐incision surgery when compared to conventional multiport laparoscopy.^41^ It seems that the risk of trocar site incisional hernia might be influenced by patient factors (obesity, pre‐existent umbilical hernia), operative factors (emergent cases, closure technique, port location), and length of follow‐up.^39^ Therefore, a proper patient and closure technique selection might help to reduce this undesired complication.

It is the opinion of the authors that the use of a rigid metallic port allows to reduce the real size of the access incision when compared to the traditional single incision compressible silicon ports. The latter require a larger incision to permit to be introduced without damaging the port. Pietrabissa et al reported a 15% silicon port rupture rate.^13^ The metallic port can be advanced stretching the fascia obtaining an overall smaller incision. The technique used for closure is also crucial to reduce the incidence of incisional hernias. From our previous robotic and laparoscopic single incision experience, we switched the closure technique from 0 Vicryl^TM^ running to 1 PDS^TM^ figure of eight interrupted stitches and this resulted in a significant decrease in the incisional hernia rate.

Remarkably, in our cohort of patients there was a high prevalence of obesity in the cholecystectomy group (62.4% of patients had a BMI above 30 and, in this group, the average BMI was 38.8 kg/m^2^ with a maximum BMI close to 72). This shows that the system is performing well in obese and superobese patients.

The SP platform still has some limitations such as the lack of advanced energy devices, staplers, and suction‐irrigation. However, we strongly believe that this platform provides significant improvements and will likely help expanding the indications and applications of single‐incision surgery.

This preliminary study has several limitations, being the lack of a control group (patients operated with other approaches) the most important. Moreover, a cost analysis was not performed as the main focus was to determine safety, feasibility, and utility of the approach. Further studies and larger series are still needed to evaluate outcomes and cost‐effectiveness of this approach.

## 6 CONCLUSIONS

Robotic SP‐C and SP‐TAPP inguinal hernia repair are safe and feasible. This platform might help to expand the applications of minimally invasive single‐incision surgery. Further studies are needed to confirm our results and to compare them to the standard laparoscopic and robotic approach.

## AUTHOR CONTRIBUTIONS

Study concept and design: All authors. Acquisition, analysis, or interpretation of data: All authors. Drafting of the manuscript: All authors. Critical revision of the manuscript for important intellectual content: All authors. Statistical analysis: Dreifuss, Chang, Bianco, Cubisino. Administrative, technical, or material support: Dreifuss, Bianco, Schlottmann, Masrur. Study supervision: Dreifuss, Bianco, Masrur, Schlottmann, Giulianotti.

## CONFLICTS OF INTEREST

Nicolas H. Dreifuss, Betty Chang, Francisco Schlottmann, Antonio Cubisino, Alberto Mangano, Yevhen Pavelko, and Mario A. Masrur have no conflicts of interest or financial ties to disclose. Bianco FM and Pier C. Giulianotti have an educational agreement with Intuitive Surgical. The University of Illinois at Chicago has an institutional agreement with Intuitive for training.

## Data Availability

The data that support the findings of this study are available from the corresponding author upon reasonable request.
